# Muscle spindle function in healthy and diseased muscle

**DOI:** 10.1186/s13395-020-00258-x

**Published:** 2021-01-07

**Authors:** Stephan Kröger, Bridgette Watkins

**Affiliations:** Department of Physiological Genomics, Biomedical Center, Ludwig-Maximilians-University Munich, Großhaderner Str. 9, 82152 Planegg-Martinsried, Germany

**Keywords:** Mechanotransduction, Sensory physiology, Proprioception, Neuromuscular diseases, Intrafusal fibers, Muscular dystrophy

## Abstract

Almost every muscle contains muscle spindles. These delicate sensory receptors inform the central nervous system (CNS) about changes in the length of individual muscles and the speed of stretching. With this information, the CNS computes the position and movement of our extremities in space, which is a requirement for motor control, for maintaining posture and for a stable gait. Many neuromuscular diseases affect muscle spindle function contributing, among others, to an unstable gait, frequent falls and ataxic behavior in the affected patients. Nevertheless, muscle spindles are usually ignored during examination and analysis of muscle function and when designing therapeutic strategies for neuromuscular diseases. This review summarizes the development and function of muscle spindles and the changes observed under pathological conditions, in particular in the various forms of muscular dystrophies.

In its original sense, the term proprioception refers to sensory information arising in our own musculoskeletal system itself [1–4]. Proprioceptive information informs us about the contractile state and movement of muscles, about muscle force, heaviness, stiffness, viscosity and effort and, thus, is required for any coordinated movement, normal gait and for the maintenance of a stable posture. Proprioception combines with other sensory systems to locate external objects relative to the body and by this contributes to our body image and equilibrioception. Since proprioception is vital for motor and body control, patients with a loss of proprioception either due to an autoimmune disease [5] or due to a loss-of-function mutation in a protein essential for proprioception [6] have prominent sensory and motor deficits, generally leading to ataxia and dysmetria. Patients with a congenital absence of proprioception show delayed development of head control and walking, an early impairment of fine motor skills, sensory ataxia with unsteady gait, increased stride-to-stride variability in force and step length, an inability to maintain balance with eyes closed (Romberg’s sign), a severely reduced ability to identify the direction of joint movements, and an absence of tendon reflexes [6–12]. The motor problems are so severe that without the compensatory activity of other senses, including the vestibular and the visual systems, the patients are unable to maintain their posture, walk or perform coordinated voluntary movements. In addition, recent studies have uncovered exciting new functions for proprioception [4, 13]. For example, proprioceptive information is required for the realignment and proper healing of fractured bones [14] as well as for the maintenance of spine alignment [15]. Thus, patients with proprioceptive deficits are likely to develop adolescent idiopathic scoliosis in their second decade of life, suggesting that the proprioceptive information may not only provide dynamic control of spine alignment but also prevent progressive spinal deformation [13, 15]. Moreover, after spinal cord injury, proprioceptive feedback is essential for locomotor recovery and facilitates circuit reorganization [16]. Ablation of this feedback after behavioral recovery permanently reverts functional improvements, demonstrating the essential role of proprioception also for maintaining regained locomotor function [17]. Thus, proprioceptive information has functions that extend far beyond motor control and includes non-conscious regulation of skeletal development and function as well as recovery after spinal cord injury [4, 18].

## Structure and function of muscle spindles

Although Golgi tendon organs, joint receptors and other sensory systems also contribute to proprioception, muscle spindles are the most important proprioceptors [19, 20]. Muscle spindles are the most frequently found sense organs in skeletal muscles and present in almost every muscle. The density of muscle spindles within the large muscle mass, however, is low so that they are rather difficult to detect. Rough estimates have suggested approximately 50,000 muscle spindles in the entire human body [21]. Interestingly, in humans, muscle spindles are mostly absent in facial muscles [22] and extraocular muscles have unusual muscle spindles and additional unique sensory structures named palisade endings, which might also provide proprioceptive information [23–25].

**Fig. 1:**
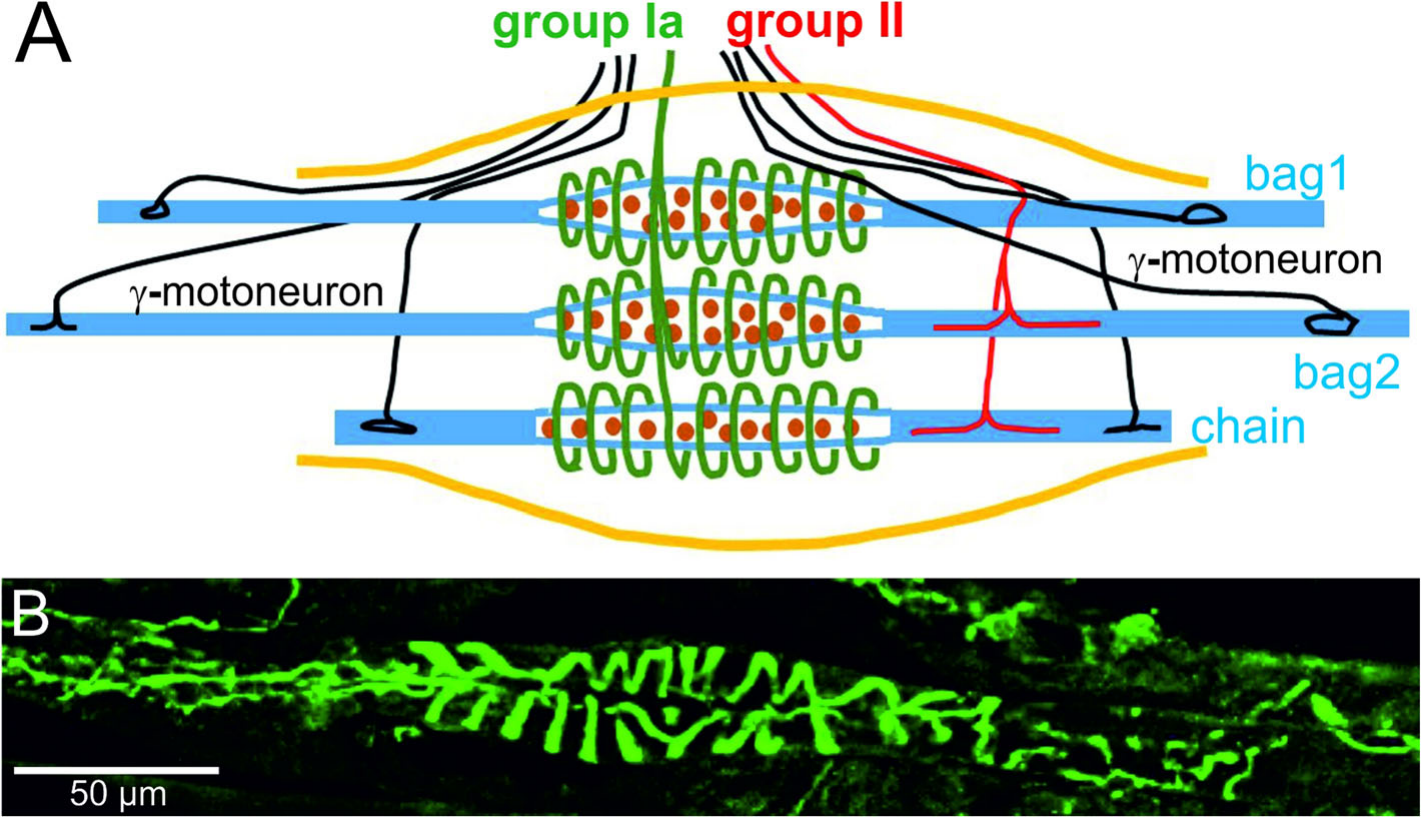
Structure of muscle spindles and distribution of the DGC. Panel **a** shows a schematic representation of the sensory and fusimotor innervation of intrafusal fibers. The connective tissue capsule is indicated in orange. Muscle spindles contain three types of intrafusal fibers: nuclear bag1, nuclear bag2, and nuclear chain fibers. Different parts of intrafusal fibers are innervated by different neurons: The central (equatorial) part is in intimate contact with afferent proprioceptive sensory neurons, termed primary “group Ia afferents” (forming the annulospiral endings) and (if present) secondary or “group II afferents”, marked in green and red, respectively. In addition to the sensory neurons, intrafusal muscle fibers are innervated by efferent γ-motoneurons (marked in black) in both polar regions, were they form a cholinergic synapse. The polar regions of intrafusal fibers contain most of the contractile elements (sarcomeres are indicated in blue in panel **a**). This schematic representation is based on the well-characterized muscle spindles from the cat’s tenuissimus muscle [19]. However, interspecies differences exist. For example, mouse muscle spindles might not have a group II innervation [26], and in humans, the sensory nerve terminal does not form annulospiral endings and the secondary ending innervates nuclear bag as well as nuclear chain muscle fibers [27]. Panel **b** shows a confocal section of the central part of a mouse muscle spindle stained with anti-neurofilament antibodies. Note the annulospiral endings of the Ia afferents in the central region. The γ-motoneuron endplates are located outside the picture

Muscle spindles are encapsulated sensory receptors which inform the brain about changes in the length of muscles [3, 20]. They consist of specialized muscle fibers (so called intrafusal fibers) that are multiply innervated and named according to the arrangement of their nuclei as nuclear bag or nuclear chain fibers (a schematic representation of a muscle spindle is shown in Fig. 1a). Intrafusal muscle fibers are up to 8-mm long in humans and about 400-μm long in mice and oriented parallel to the surrounding (extrafusal) muscle fibers. Each muscle spindle contains on average 3–5 (mouse) [28] or 8–20 (human) [29] intrafusal fibers. With a diameter of 8 to 25 μm [30], intrafusal muscle fibers are much thinner than extrafusal muscle fibers. Contractile filaments are found in intrafusal fibers predominantly in the polar regions with only a small ring of sarcomeres underneath the sarcoplasmic membrane in the central (equatorial) region (Fig. 1a). However, muscle spindles do not contribute significantly to the force generated by the muscle [31, 32]. Nuclear bag fibers often extend beyond the fluid-filled fusiform capsule and are attached to intramuscular connective tissue [33]. Nuclear chain fibers are attached to the polar regions of the thicker and longer nuclear bag fibers [33].

Functionally, muscle spindles are stretch detectors, i.e. they sense how much and how fast a muscle is lengthened or shortened [19]. Accordingly, when a muscle is stretched, this change in length is transmitted to the spindles and their intrafusal fibers which are subsequently similarly stretched. To respond appropriately to changes in muscle fiber length, intrafusal fibers are innervated by two kinds of neurons: afferent sensory neurons and efferent motoneurons (Fig. 1a). In humans, the sensory innervation of the muscle spindle arises from both group Ia and group II afferent fibers (also sometimes called type Ia or type II fibers, respectively), which differ in their axonal conduction velocity [34]. In contrast, in mice an innervation by group II fibers has so far not been detected by histological or functional assays [26, 35]. However, transcriptome analysis of DRG proprioceptive neurons has recently suggested the existence of group II fibers also in mice [36]. There is usually only a single Ia afferent fiber per spindle, and every intrafusal muscle fiber within that spindle receives innervation from that sensory neuron. In cat, rat and mice (and probably many other species), the axon terminals of this sensory afferent fiber coil around the central (equatorial) part of both nuclear bag and nuclear chain fibers, forming the primary endings (also called annulospiral endings) [37, 38] (Fig. 1b). In humans, sensory terminals form irregular coils with branches and varicose swellings [39]. When present, the smaller group II fiber terminals flank the primary annulospiral endings in the equatorial region (Fig. 1a). There may be several group II fibers innervating each human spindle [40]. The cell bodies of these proprioceptive afferent fibers constitute 5–10% of all neurons in the dorsal root ganglion [36]. They can be classified and distinguished from other dorsal root ganglion neurons as a unique neuronal population using single cell transcriptome analysis [36, 41, 42].

**Fig. 2:**
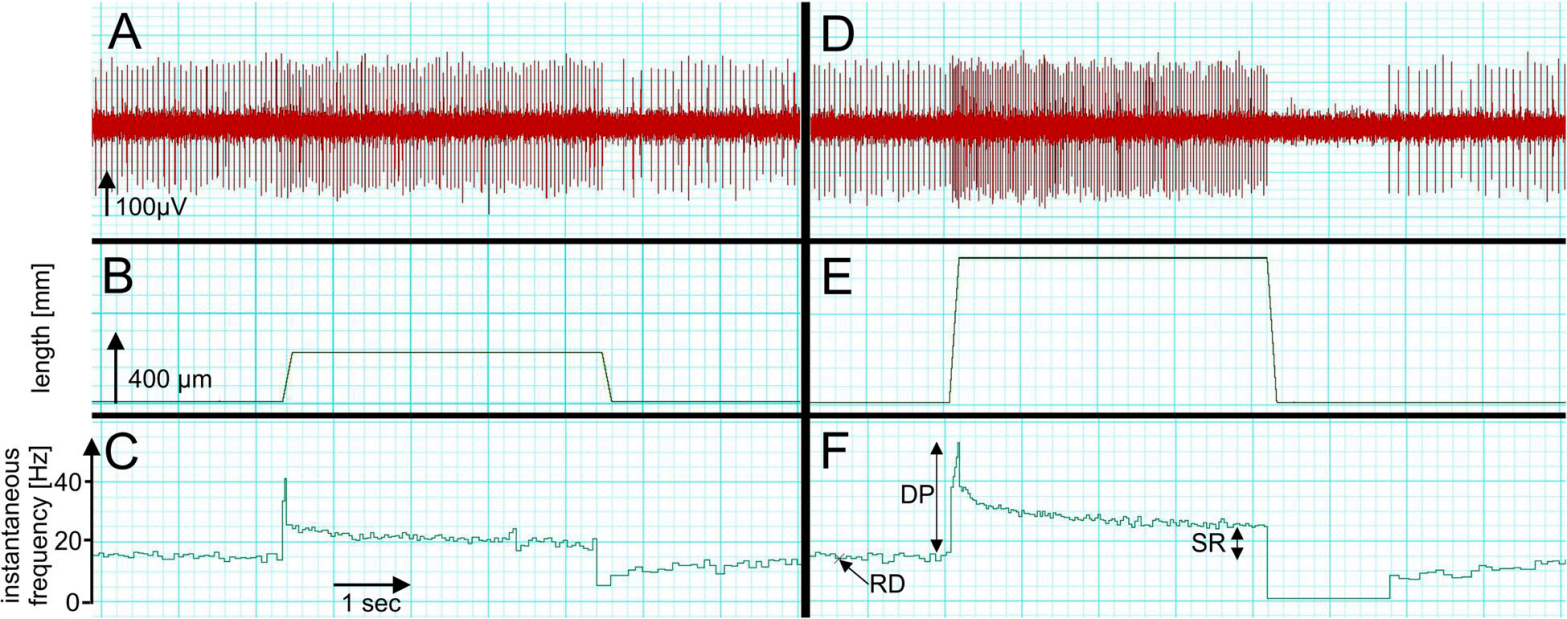
Typical responses of a muscle spindle to stretch. The responses of an individual muscle spindle from the mouse extensor digitorum longus muscle to ramp and hold stretches applied to the tendon were recorded with an extracellular electrode. Single unit action potentials are shown in (**a** and **d**). The stretch was 4-s long, and the length change corresponded to 260 (panel **b**) and 780 (panel **e**) μm. The ramp speed in (**e**) was 3-fold higher compared to that in (**b**). Panels **c** and **f** represent the instantaneous frequencies (action potentials/s). In panel **f**, three different parameters that are usually analyzed to describe muscle spindle function are illustrated: resting discharge (RD), dynamic peak (DP), and static response (SR). For more information on these parameters, see [32, 33, 44]. Note that the dynamic peak and the static response is higher in (**f**), compared in (**c**) due to the higher ramp speed and the longer length change. Since the fusimotor innervation was cut during the dissection of the muscle, no action potentials can be observed directly after the end of the ramp and hold stretch (spindle pause)

Afferent sensory neurons generate action potentials with frequencies that correspond to the size of the stretch and to the rate of stretching [43] (Fig. 2). Sensory neurons innervating bag1 fibers respond maximally to the velocity of changes in muscle fiber length (dynamic sensitivity) and those innervating bag2 fibers as well as nuclear chain fibers respond maximally to the amount of stretch (static sensitivity). For a recent review on the mechanotransduction processes within the sensory nerve terminal, see [45].

Sensory neuron activity from muscle spindles can be electrophysiologically recorded and characterized in a number of different ways. In humans, for example, individual sensory afferent (“single unit”) action potentials can be studied in vivo by intraneural microelectrodes inserted into accessible peripheral nerves (microneurography), such as the median and ulnar nerves at the wrist or upper arm, the radial nerve in the upper arm, and the tibial and common peroneal nerves in the lower limb [29]. In mice, single unit muscle spindle afferent responses to ramp-and-hold stretches and sinusoidal vibratory stimuli have been well characterized in an ex vivo adult mouse extensor digitorum longus preparation dissected with the innervating nerve attached [26, 46]. A typical example for a single unit muscle spindle response to two different ramp-and-hold stretches in the adult mouse extensor digitorum longus muscle is shown in Fig. 2. In many species, muscle spindles exhibit a resting discharge that is related to the degree of muscle stretch but the frequency of the mean firing rate differs between species. In mice at room temperature, the frequency is ~ 15 Hz (Fig. 2). Muscle spindle afferents encode muscle length in their frequency of firing, i.e. the more the muscle is stretched, the higher the frequency (static response). In addition to the static encoding of length changes, spindle afferents, especially primary afferents, can respond to dynamic length changes, i.e. the faster the stretch, the higher the frequency during the ramp phase. Accordingly, the instantaneous frequency (action potentials/s) shown in Fig. 2 is higher the faster the stretch is and the longer the length change is.

In addition to sensory neurons, intrafusal muscle fibers are also innervated by efferent motoneurons (fusimotor innervation; Fig. 1a) [47]. Both β- and γ-motoneurons innervate intrafusal fibers, but γ-motoneurons are considerably more abundant and much better characterized compared to β-motoneurons [48]. Gamma-motoneurons constitute about 30% of all motoneurons in the ventral horn of the spinal cord. Axons of motoneurons usually enter the spindle together with the sensory fibers in the central region but innervate intrafusal muscle fibers exclusively in the polar regions. The endplates of γ-motoneurons differ structurally from the neuromuscular junctions formed by α-motoneurons on extrafusal fibers, but both are cholinergic synapses with many features in common, including junctional folds and a basal lamina filling the synaptic cleft [47]. Moreover, both synapses require the extracellular matrix synapse organizer agrin and its receptor complex (consisting of the low-density lipoprotein receptor-like protein 4 and the tyrosine kinase MuSK) for their formation, suggesting a common molecular basis for their synaptogenesis [49]. Gamma-motoneurons induce contractions of sarcomeres in the polar region to exert tension on the central region of intrafusal fibers [47, 50]. This prevents the slackening of intrafusal fibers during muscle shortenings and allows for continuous adjustment of the mechanical sensitivity of spindles over the wide range of muscle lengths and stretch velocities that occur during normal motor behaviors.

## Muscle spindle development and ageing

**Fig. 3:**
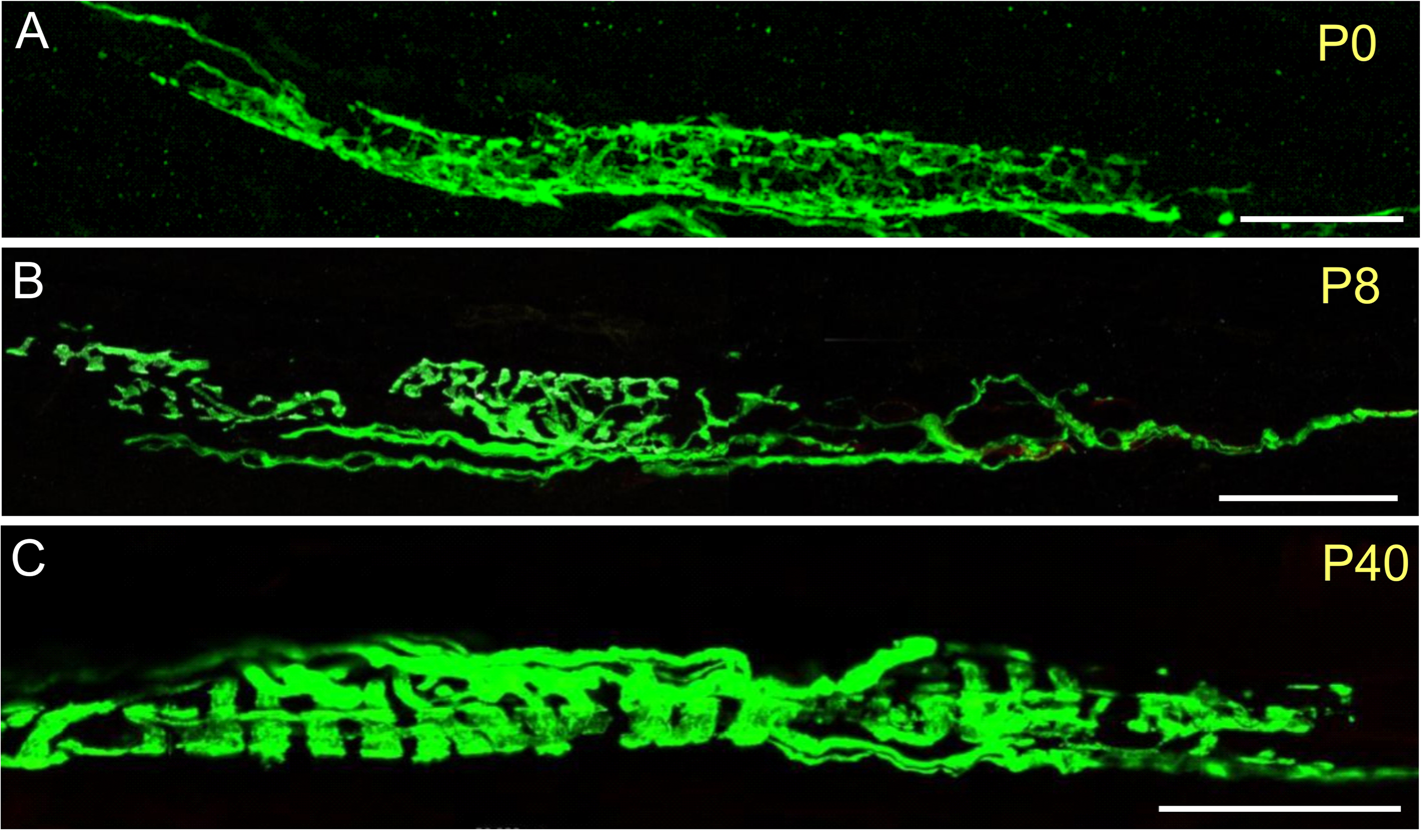
Postnatal development of mouse muscle spindles. Muscle spindles from postnatal day 0: P0 (**a**), P8 (**b**), and P40 (**c**). Thy1-YFP mouse extensor digitorum longus were stained with anti-GFP antibodies. Only the central (equatorial) region is shown. Note the transformation of the “web-like” appearance of the sensory nerve terminal into the typical annulospiral ending during postnatal development. Scale bar in all panels: 50 μm

Muscle spindle development starts during embryonic stages but continues well into adult life [51]. Human muscle spindles can be recognized in fetal tissue around the 11th week of gestation [52, 53], but little is known about the molecular basis of human muscle spindle development. In contrast, muscle spindle development is much better characterized in rodents, where muscle spindle differentiation begins around embryonic day 14 when the growth cone of the sensory neuron’s axon reaches its target muscle. Fusimotor innervation develops a few days later and is present in mice at E19 [54]. In rodents and humans, immature myotubes are induced to differentiate into intrafusal fibers when sensory afferent axons contact the primary myotubes [55–57]. Apparently, nuclear bag fibers differentiate before nuclear chain fibers in rats [58, 59]. There is the possibility of a hyperinnervation of intrafusal fibers with subsequent pruning of the terminals for the fusimotor innervation [60] as well as for the sensory innervation [61] of rat muscle spindles. In mice, the intrafusal fibers are initially surrounded by a “web-like” network of sensory axons, which is reduced to an adult primary ending from a single sensory neuron (Fig. 3). Human muscle spindles are functional at birth, but their response to stretch is immature [30]. Moreover, with the postnatal increase in muscle mass and mobility, sensory nerve terminals in mice and humans undergo a number of anatomical and physiological changes [62–64]. By postnatal day 18, muscle spindle afferent firing is indistinguishable from the firing in adult rats suggesting that muscle spindle maturation continues into postnatal life and that muscle spindles are capable of responding to stretch, even at an age when their morphological and ultrastructural maturation is not yet fully accomplished [65].

After the establishment of a physical contact between the sensory axon and the primary myotube, both cells exchange inductive signals ensuring the differentiation of intrafusal fiber and the survival of the sensory neuron. This reciprocal signaling is essential for muscle spindle differentiation and intrafusal fiber development. Accordingly, elimination of the sensory input (but not of the fusimotor input) in embryonic and adult muscle spindles results in a rapid degeneration of the intrafusal fibers ([66–68]; for review, see [55]). The key inductive factor for the sensory neuron-mediated muscle spindle differentiation is the immunoglobulin form of neuregulin-1 (Ig-Nrg1). Ig-Nrg1 is expressed by proprioceptive neurons [69, 70], and its release from sensory neurons and subsequent binding to the ErbB2 receptor expressed by immature muscle fibers [71] induces their differentiation into intrafusal muscle fibers. Accordingly, Nrg1- or ErbB2-deficient mice do not initiate muscle spindle differentiation, do not elaborate Ia afferent terminals and have an ataxic behavior as well as abnormal hind limb reflexes, consistent with severe proprioceptive deficits [69–72]. Nrg1–ErbB2 signaling activates downstream targets such as the transcription factor early growth response protein 3 (*Egr3*) [73–75], and the Ets transcription factors *Pea3*, *Erm* and *Er81* as well as the Grb2-associated binder 1 protein, a scaffolding mediator of receptor tyrosine kinase signaling [69, 76, 77]. Although muscle spindles are initially generated in *Egr3*-deficient mice [75], subsequently most of them degenerate, resulting in ataxic behavior [73, 74]. Overexpression of *Egr3* in primary myotubes on the other hand leads to their differentiation into intrafusal fibers [78], suggesting that this transcription factor is necessary and sufficient for muscle spindle maintenance. Interestingly, Ig-Nrg1 is the substrate for the membrane-bound aspartyl protease Bace1 (also called β-secretase 1). Cleavage of Ig-Nrg1 is required for Ig-Nrg1 function and, accordingly, in the absence of Bace1, muscle spindle numbers are reduced and spindle maturation is impaired. Moreover, a graded reduction in Ig-Nrg1 signal strength in vivo induced by pharmacological Bace1 inhibition results in increasingly severe deficits in the formation and maturation of muscle spindles in combination with a reduced motor coordination [70]. The continuous presence of Bace1 and Ig-Nrg1 is essential to maintain muscle spindles in adult muscle, since either pharmacological inhibition of Bace1 or induced Bace1 deficiency in adult proprioceptive neurons also leads to a decline of muscle spindle number [70]. In summary, the sensory neuron induces the differentiation of muscle spindles from immature myotubes via Ig-Nrg1, Bace1 and ErbB2-mediated activation of Egr3.

On the other hand, muscle fibers release neurotrophin-3 (NT3), which activates the tropomyosin receptor kinase C (TrkC) receptor on proprioceptive sensory neurons and by this secures the survival of the sensory neuron [79–81]. The TrkC/NT3 signaling system is, however, not required for the initiation of muscle spindle differentiation [82]. Muscle-specific overexpression of NT3 results in an increase in the number of proprioceptive afferents and muscle spindles [83–85]. NT3/TrkC signaling induces the expression of the *Etv1* (*Er81*) transcription factor in proprioceptive sensory neurons [76, 86]. Interestingly, the survival of proprioceptive sensory neurons supplying distinct skeletal muscles differ in their dependence on *Etv1* for their survival and differentiation [87]. The survival and/or specification of the TrkC-positive proprioceptive afferents also requires the expression of the Runt-related transcription factor 3 (*Runx3*) and *Runx3*-knockout mice display severe limb ataxia due to absence of proprioceptive sensory neurons [88, 89].

As in the musculoskeletal system in general, various elements of the proprioceptive system decline during ageing [90, 91]. These changes might contribute to the frequent falls and motor control problems observed in older adults. On the structural level, muscle spindles in aged humans possess fewer intrafusal fibers, an increased capsular thickness and some spindles which show signs of denervation [92, 93]. In old rats, primary endings are less spiral or non-spiral in appearance, but secondary endings appeared unchanged [94, 95]. Likewise, in old mice, there is a significant increase in the number of Ia afferents with large swellings that fail to properly wrap around intrafusal muscle fibers. There is also a degeneration of proprioceptive sensory neuron cell bodies in the dorsal root ganglion but no change in the morphology and number of intrafusal muscle fibers [96]. In addition, electrophysiological studies showed that mature rat muscle spindles display a lower dynamic response of primary endings compared with those of young animals [94]. Taken together, the proprioceptive system undergoes significant structural and functional changes with advancing age and the changes are consistent with a gradual decline in proprioceptive function in elderly individuals and animals.

## Muscle spindle structure and function in muscular dystrophy

An impaired proprioception, in some cases associated with an altered muscle spindle morphology, has been documented as a secondary effect in many diseases. These include Parkinson’s disease [97], Huntington’s disease [98], multiple sclerosis [99], Charcot-Marie-Tooth type 2E [100], traumatic or neurotoxic injury [101], spinal muscular atrophy [102], diabetic neuropathy [103, 104] and myasthenia gravis [105, 106]. In amyotrophic lateral sclerosis, sensory neurons are similarly affected as α-motoneurons [107–110]. They accumulate misfolded SOD1 protein and the annulospiral endings degenerate, leading to ataxia and motor control problems [107, 109]. In contrast to α-motoneurons, γ-motoneurons apparently survive degeneration in murine models of amyotrophic lateral sclerosis and spinal muscular atrophy [111–113], suggesting differential vulnerabilities for both types of motoneurons in both diseases.

Recently, a number of studies have analyzed proprioception and muscle spindle function in patients with muscular dystrophy and in dystrophic mouse models. Muscular dystrophies are a heterogeneous group of more than 30 different mostly inherited diseases characterized by muscular weakness and atrophy in combination with degeneration of the musculoskeletal system [114]. The molecular basis of many muscular dystrophies are mutations that directly or indirectly influence the function of the dystrophin-associated glycoprotein complex (DGC) [115, 116]. The most common form of muscle dystrophy in humans is Duchenne muscular dystrophy (DMD) which affects approximately 1 in 5000 boys [117]. DMD is caused by mutations in the *DMD* gene, which codes for the large cytoskeletal protein dystrophin [114]. In skeletal muscle, dystrophin links subsarcolemmal F-actin filaments to the extracellular matrix via the DGC [118, 119]. This link mechanically stabilizes the sarcolemmal membrane particularly during muscle contraction. Mutations which cause an interruption of the dystrophin/DGC-mediated molecular connection lead to mechanical lability of the sarcolemmal membrane and subsequent contraction-induced damage [114, 120–122]. While regeneration of damaged muscle fibers occurs initially, it cannot compensate for the prolonged degenerative loss of muscle tissue [123], leading over time to a reduction of muscle mass, loss of contractile force and, in the case of DMD, to premature death of the affected person due to respiratory or cardiac muscle failure [124].

Many muscular dystrophy patients suffer from postural instability, sudden spontaneous falls and poor manual dexterity [125–128], suggesting that their proprioceptive system might be impaired. However, only minor morphological changes in muscle spindles were detected in human dystrophic muscles. These changes primarily affect the connective tissue surrounding intrafusal fibers. For example, thickening of the capsule and of the connective tissue septa inside the spindle and an “oedematous swelling” of the spindle were reported in muscle biopsy specimens from Duchenne- and limb-girdle muscular dystrophy patients [106]. Likewise, analyses of biopsy specimens from patients with muscular dystrophy and with congenital dystrophy revealed an increased thickness of the spindle capsule and a slight decrease of the intrafusal fiber diameter [129]. An autopsy study of seven DMD patients aged 15 to 17 years reported more severe pathological changes including degenerative changes, atrophy and loss of intrafusal muscle fibers [130], but it is unclear if these more extensive changes were caused by the disease or due to postmortem tissue degeneration. This possibility has to be considered, since proprioceptive functions of muscle spindles in DMD patients appear rather normal (see below) and since a recent study analyzing muscle spindles from a 27-year-old severely affected DMD patient described that spindle size and number as well as the size of intrafusal myofibers and capsule thickness were in the normal range [131]. Interestingly, the extrafusal fibers directly surrounding the muscle spindles were also less affected by the degenerative events compared to fibers further away from the spindle, suggesting the possibility of a more protective environment directly around muscle spindles.

Likewise, murine models for several muscular dystrophies display only minor changes in muscle spindle structure compared to wildtype control mice. For example, muscle spindles in the soleus muscle from 1-year-old C57BL/6J^dy-2J/dy-2J^ (Lama2^dy2J/dy2J^) dystrophic mice, a model for laminin α2 (merosin)-deficient congenital muscular dystrophy, had a small but significant increase in the diameter of the outer capsule and in the overall thickness of the equatorial region [132]. But, as in the corresponding patients, intrafusal fibers and sensory terminals appeared mostly spared from degeneration [44, 132]. Similarly, the DMD^*mdx*^ mouse line [133], a widely used model system for muscular dystrophy of the Duchenne type [134], revealed no reduction of the total number of muscle spindles and no change in the structure of muscle spindles and their sensory innervation [135, 136]. Thus, compared to extrafusal muscle fibers, the morphology of intrafusal muscle fibers and of muscle spindles generally appear much less affected by the degenerative processes in humans and in mice with Duchenne-type muscular dystrophy.

The mechanism(s), which protect intrafusal myofibers from degeneration and wasting, are unknown. Capsular thickening in the equatorial region may be an adaptive response, preventing the intrafusal fibers from undergoing atrophy. Another explanation for the sparing of muscle spindles in DMD patients could be a better maintenance of the intracellular calcium homeostasis similar to what has been described for extraocular muscles [137]. Furthermore, the mild phenotypic effect of the dystrophin mutations might be due to the different surface-to-volume ratio, compared to extrafusal fibers. Intrafusal fibers are thinner compared to extrafusal fibers, have a much smaller mechanical burden, and generate considerably less contractile force. They are therefore less likely to suffer from mechanical damage [138].

**Fig. 4:**
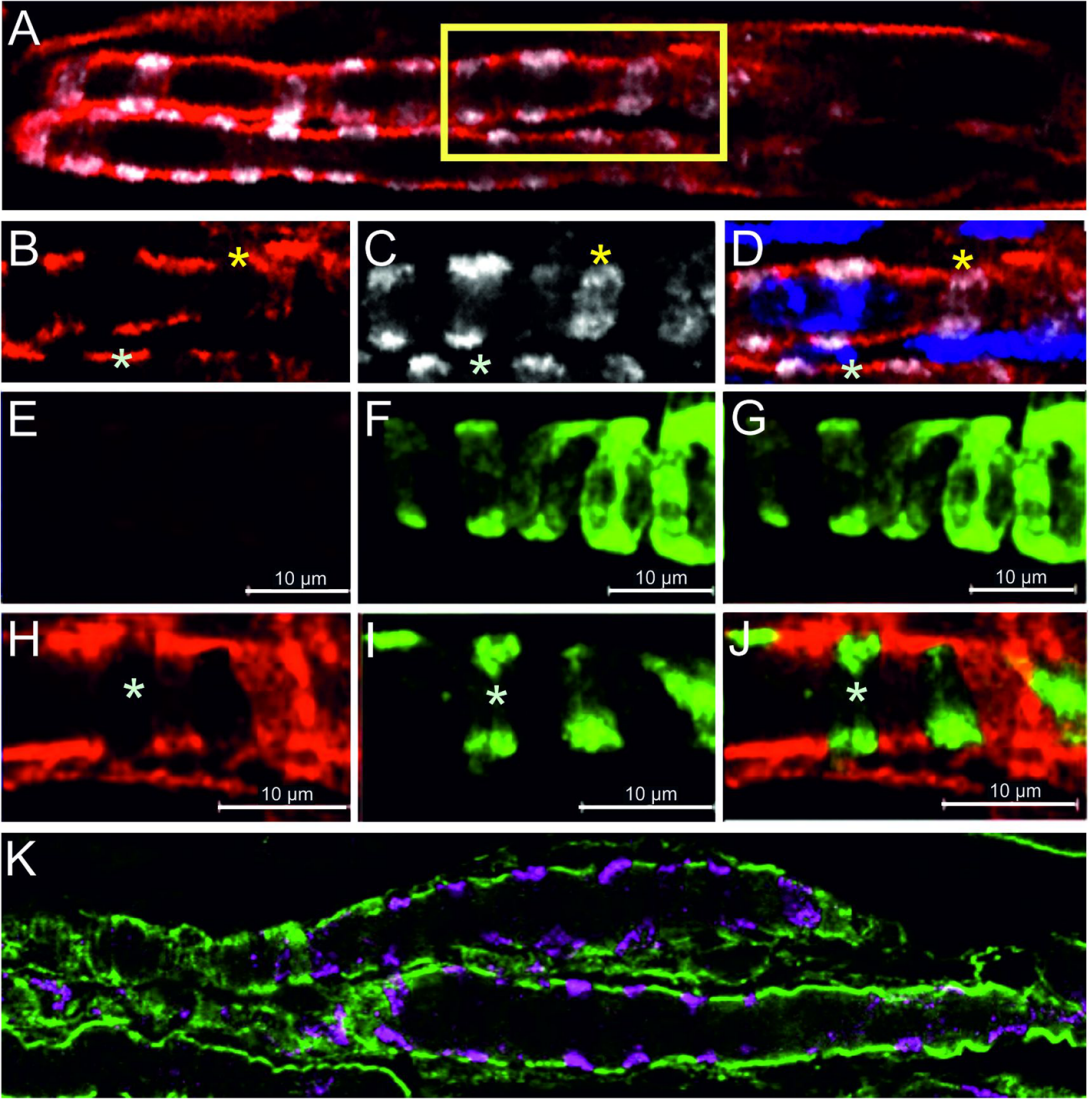
Distribution of the dystrophin glycoprotein complex in mouse intrafusal fibers. Panel **a** shows two intrafusal fibers labeled by anti-dystrophin antibodies (red channel) and by antibodies against the vesicular glutamate transporter 1 (vGluT1; white channel). Panels **b**–**d** show the boxed area in panel **c** at a higher magnification. Note that dystrophin is concentrated in the intrafusal fiber plasma membrane in areas that are not in contact with the sensory neuron. The blue color represents nuclei stained with 4′,6-diamidin-2-phenylindol (DAPI). Panels **e–j** show the distribution of utrophin (red channel) in the central region of muscle spindles from wildtype (**e–g**) and from DMD^*mdx*^ mice (**h–j**). Anti-vGluT1 antibodies (green channel in panels **e–j**) were used to label the sensory nerve terminal. Panels **d**, **g** and **j** show the merged channels. Utrophin is not detectable in the equatorial region of muscle fibers from wildtype mice (**e**) but severely upregulated in intrafusal fibers from DMD^*mdx*^ mice (**h**). Note the absence of utrophin in the contact area between intrafusal fiber and sensory nerve terminal. Asterisks mark corresponding positions in all panels. Panel **k** shows a single confocal section of a muscle spindle stained with antibodies against vGluT1 (magenta) and against dystrobrevin (green) to indicate that other components of the DGC have a similar distribution as dystrophin, i.e. are concentrated in areas of the intrafusal fiber that are not in contact with the sensory nerve terminal

Immunohistochemical analysis showed that dystrophin is present in the sarcolemma of the polar regions of intrafusal fibers [139]. In contrast, in the equatorial region, dystrophin is absent from that part of the intrafusal fiber, which is in contact with the sensory nerve terminal but concentrated in parts without sensory nerve contact [136, 139] (Fig. 4a–d). Other proteins of the DGC (including alpha-dystrobrevin1; Fig. 4k) have a similar distribution. The area, where the DGC is concentrated, also corresponds to the region where the intrafusal fiber has direct contact to the basal lamina. The interaction of DGC components with basal lamina proteins might stabilize and help to maintain the subcellular concentration of the DGC in this region of the intrafusal fiber. In any case, the unusual distribution of DGC components indicates a molecular specialization in particular regions of the intrafusal fiber plasma membrane.

As expected, dystrophin is absent in intrafusal fibers of DMD^*mdx*^ mice [136] (Fig. 4e–g). However, utrophin expression is markedly upregulated and has a similar distribution in DMD^*mdx*^ mice as dystrophin in wildtype mice [136] (Fig. 1e–j). Utrophin is an autosomally encoded paralogue of dystrophin [136]. It shares more than 80% amino acid sequence similarity to dystrophin, has a similar domain structure and like dystrophin can interact with actin filaments and with DGC components [140]. In skeletal muscle, utrophin is highly expressed in fetal and regenerating muscle fibers [141, 142]. In adult wildtype muscle fibers, utrophin is replaced by dystrophin along the entire sarcolemmal membrane but remains present at the neuromuscular junction, the myotendinous junction and blood vessels [143–146]. In extrafusal muscle fibers from DMD^*mdx*^ mice, utrophin is greatly upregulated and present along the entire sarcolemma [147, 148]. The upregulation of utrophin expression in extrafusal muscle fibers can lessen or even prevent the dystrophic phenotype in DMD^*mdx*^ mice and muscular dystrophy patients [149–153]. The upregulation of utrophin in intrafusal fibers of DMD^*mdx*^ mice might therefore functionally compensate for the absence of dystrophin and prevent the degeneration of intrafusal fibers. However, intrafusal muscle fibers from DMD patients are utrophin-negative [131], suggesting that the upregulation of this protein cannot solely explain the preservation of intrafusal muscle fibers in humans.

An obvious question arising from these observations is whether the relatively minor structural changes in muscle spindles from DMD patients and corresponding mouse models are accompanied by functional changes. Analysis of single unit sensory afferent recordings from DMD^*mdx*^ mice showed that muscle spindles have a normal response to ramp-and-hold stretches and only a slightly increased response to sinusoidal vibrations [136]. More strikingly, the resting discharge, i.e. the action potential frequency of sensory afferents from a resting muscle spindle (Fig. 2), was significantly increased in DMD^*mdx*^ mice compared to control mice. This increase in the resting discharge might be clinically relevant since it would cause an increased muscle tone via the muscle stretch reflex, which would lead to an increase in muscle stiffness and an aggravation of the degenerative events in extrafusal fibers of DMD patients.

Interestingly, a similar increase of the resting discharge was observed in SJL-Dysf C57BL/6 (*dysf*^−/−^) mice [136], a murine model system for dysferlinopathies [154, 155]. Dysferlinopathies (including limb girdle muscular dystrophy 2B and Miyoshi myopathy) are muscular dystrophies characterized by muscle weakness and wasting but differ from DMD in the molecular etiology and disease progression [156]. They are caused by mutations in the *DYSF* gene that impair the function of dysferlin [157–159], a single pass transmembrane protein with important roles in membrane fusion and trafficking [156, 160, 161]. When microlesions in the plasma membrane occur, vesicles are recruited to the injury site and dysferlin then appears to participate in the resealing of the injury site by promoting vesicle aggregation and fusion with the plasma membrane [162, 163]. Accordingly, loss of dysferlin leads to an impaired membrane repair and degeneration of skeletal muscle fibers, causing the muscle weakness. Additional functions of dysferlin, including an impaired Ca^2+^ homeostasis during mechanical stress [164], might contribute to the degeneration of skeletal muscle. Like in the DMD^*mdx*^ mouse, muscle spindle number and morphology of intrafusal fibers and their innervation were not changed, but the resting discharge frequency was increased qualitatively and quantitatively similar to DMD^*mdx*^ mice [136]. The similarity of the functional changes in DMD^*mdx*^ and *dysf*^−*/*−^ mice suggests a common deficit in both mouse strains, but the molecular mechanism is unknown. The double-mutant mice did not have an aggravated phenotype, suggesting that both mutations coalesce on the same pathway [136].

In contrast to the functional changes in murine model systems for different forms of muscular dystrophy, little if any functional deficits have been observed in muscular dystrophy patients. For example, muscular dystrophy patients perceive passive movements, experience illusory movement induced by muscle tendon vibration, and have proprioceptive-regulated sways in response to vibratory stimulation applied to the neck and ankle muscle tendons [165]. Moreover, reinforcement maneuvers increased the sensitivity of muscle spindle afferents to imposed movements of the ankle in a similar way in DMD patients and in non-affected controls [166]. These findings argue for either preserved proprioceptive functions of muscle spindles or the activation of compensatory mechanisms.

The morphological phenotype in Duchenne muscular dystrophy is rather mild, but are considerably more severe in muscle spindles from patients with myotonic dystrophy, where extensive intrafusal fiber splitting was reported [167, 168]. In addition, sensory endings were undetectable on nuclear bag and nuclear chain fibers. In agreement with these pronounced ultrastructural changes, areflexia has been reported in myotonic dystrophy [169], congenital dystrophies [170] and centronuclear myopathy [171], but not in patients with tibial muscular dystrophy [172].

In summary, studies in humans and mice with muscular dystrophies show various degrees of impairment of muscle spindle function and proprioception. The deficits could alter joint coordination, impair movements and contribute to the instable gait, frequent falls and motor control problems of muscular dystrophy patients. Caregivers and patients should therefore consider an impaired proprioception when developing guidelines and when testing new interventions.

## Therapeutic strategies to improve muscle spindle function and proprioception

The most prevalent symptom of all muscular dystrophy patients is the loss and wasting of skeletal muscle tissue. Therefore, common therapeutic interventions for patients with muscular dystrophy must aim at increasing muscle strength and reducing muscle fatigue and degeneration. A proprioceptive impairment is certainly not the sole cause for the motor control problems in these patients, but the important role of the sensory system controlling motor coordination should not be ignored. In any neuromuscular disease, therapeutic strategies should therefore also aim at restoring/maintaining proprioception and muscle spindle function.

Several ways of improving muscle spindle function in dystrophic patients can be envisioned. The recent identification of the Piezo2 channel as the primary mechanotransduction channel [6, 173] might be exploited to develop drugs, which specifically target mechanosensitivity without interfering with extrafusal muscle fiber function or with neuromuscular transmission [174]. These drugs could either directly affect the Piezo2 channel [175] or indirectly, for example via modulatory Gi-coupled receptors [176]. However, potential drugs still await clinical trials and approval and side effects due to interference with Piezo2 channels in non-muscle tissues might limit their application [174].

Alternatively, training of the proprioceptive sense is a valuable behavioral therapy for improving impaired motor function and can significantly improve motor control dysfunctions in many neuromuscular disorders and in aging-related proprioceptive decline [177]. Specific proprioceptive training can improve balance control [178], motor learning [177] and walking parameters [179]. A vibratory-based proprioceptive training has been successfully used during rehabilitation to reduce the decline of motor control in subjects with facioscapulohumeral muscular dystrophy [180] and with Parkinson patients [181]. In muscular dystrophy patients, this training slows down the deleterious effects of the gradual decline of motor abilities [166]. Since muscle spindle afferent firing is modified by the emotional context [182], it is conceivable to exploit the emotional situation and vibrational stimuli during physical rehabilitation or training to increase proprioceptive acuity.

Finally, muscle spindle preservation in DMD may be an important factor to exploit new therapeutic approaches for muscular dystrophy patients. For example, the strong upregulation of the utrophin expression in intrafusal fibers from DMD^*mdx*^ mice [136] might be used to investigate the regulation of the utrophin expression in more detail. Since utrophin can functionally compensate dystrophin deficiency, a better understanding of the signaling cascade underlying utrophin upregulation in DMD^*mdx*^ mice might aid in developing strategies for a pharmacological or genetic activation of utrophin expression [183], which might also be applicable to upregulate utrophin expression in extrafusal fibers.

In summary, therapeutic strategies for muscular dystrophy patients should include in addition to strengthening the contractile muscle force, the preservation of muscle spindles and the sensitization of proprioception in order to maintain appropriate motor control and a stable gait and posture.

## Data Availability

Not applicable.

## Abbreviations

CNS: Central nervous system
DAPI: 4′,6-Diamidin-2-phenylindol
DGC: Dorsal root ganglion
DMD: Duchenne muscular dystrophy
DP: Dynamic peak
ErbB2: Erb-b2 receptor tyrosine kinase 2
Egr3: Early growth response protein 3
Etv1: ETS variant transcription factor 1
Ig-Nrg1: Immunoglobulin form of neuregulin-1
MuSK: Muscle-specific kinase
NT3: Neurotrophin-3
P: Postnatal day
RD: Resting discharge
Runx3: Runt-related transcription factor 3
SR: Static response
TrkC receptor: Tropomyosin receptor kinase C receptor
vGluT1: Vesicular glutamate transporter 1
